# c-Fms-mediated differentiation and priming of monocyte lineage cells play a central role in autoimmune arthritis

**DOI:** 10.1186/ar2940

**Published:** 2010-02-24

**Authors:** Ricardo T Paniagua, Anna Chang, Melissa M Mariano, Emily A Stein, Qian Wang, Tamsin M Lindstrom, Orr Sharpe, Claire Roscow, Peggy P Ho, David M Lee, William H Robinson

**Affiliations:** 1 Department of Medicine, Division of Immunology and Rheumatology, Stanford University School of Medicine, CCSR 4135, 269 Campus Drive, Stanford, CA 94305, USA; 2 GRECC, Palo Alto VA Health Care System, 3801 Miranda Avenue, Palo Alto, CA 94304, USA; 3 Department of Neurology and Neurological Sciences, Stanford University School of Medicine, Beckman B-002, 279 Campus Drive, Stanford, CA 94305, USA; 4 Department of Medicine, Division of Rheumatology, Immunology and Allergy, Brigham and Women's Hospital, Harvard Medical School, 1 Jimmy Fund Way, Smith 552B, Boston, MA 02115, USA

## Abstract

**Introduction:**

Tyrosine kinases are key mediators of multiple signaling pathways implicated in rheumatoid arthritis (RA). We previously demonstrated that imatinib mesylate--a Food and Drug Administration (FDA)-approved, antineoplastic drug that potently inhibits the tyrosine kinases Abl, c-Kit, platelet-derived growth factor receptor (PDGFR), and c-Fms--ameliorates murine autoimmune arthritis. However, which of the imatinib-targeted kinases is the principal culprit in disease pathogenesis remains unknown. Here we examine the role of c-Fms in autoimmune arthritis.

**Methods:**

We tested the therapeutic efficacy of orally administered imatinib or GW2580, a small molecule that specifically inhibits c-Fms, in three mouse models of RA: collagen-induced arthritis (CIA), anti-collagen antibody-induced arthritis (CAIA), and K/BxN serum transfer-induced arthritis (K/BxN). Efficacy was evaluated by visual scoring of arthritis severity, paw thickness measurements, and histological analysis. We assessed the *in vivo *effects of imatinib and GW2580 on macrophage infiltration of synovial joints in CIA, and their *in vitro *effects on macrophage and osteoclast differentiation, and on osteoclast-mediated bone resorption. Further, we determined the effects of imatinib and GW2580 on the ability of macrophage colony-stimulating factor (M-CSF; the ligand for c-Fms) to prime bone marrow-derived macrophages to produce tumor necrosis factor (TNF) upon subsequent Fc receptor ligation. Finally, we measured M-CSF levels in synovial fluid from patients with RA, osteoarthritis (OA), or psoriatic arthritis (PsA), and levels of total and phosphorylated c-Fms in synovial tissue from patients with RA.

**Results:**

GW2580 was as efficacious as imatinib in reducing arthritis severity in CIA, CAIA, and K/BxN models of RA. Specific inhibition of c-Fms abrogated (i) infiltration of macrophages into synovial joints of arthritic mice; (ii) differentiation of monocytes into macrophages and osteoclasts; (iii) osteoclast-mediated bone resorption; and (iv) priming of macrophages to produce TNF upon Fc receptor stimulation, an important trigger of synovitis in RA. Expression and activation of c-Fms in RA synovium were high, and levels of M-CSF were higher in RA synovial fluid than in OA or PsA synovial fluid.

**Conclusions:**

These results suggest that c-Fms plays a central role in the pathogenesis of RA by mediating the differentiation and priming of monocyte lineage cells. Therapeutic targeting of c-Fms could provide benefit in RA.

## Introduction

Rheumatoid arthritis (RA) is an autoimmune synovitis that affects 0.6% of the world population [1]. RA is characterized by inflammation and pannus formation in the synovial joints and by periarticular erosions, biomechanical dysfunction, and early mortality. Although the advent of biological therapeutics has revolutionized the treatment of RA, a significant number of patients with RA do not respond well to therapy. The current generation of biologic agents either blocks a critical cytokine, such as tumor necrosis factor (TNF) [2], or targets cells of the adaptive immune system, such as B [3] and T [4] cells. However, non-antigen-specific cellular responses may also contribute to the pathogenesis of RA [1]. While adaptive autoimmune responses directed against synovial joint antigens are likely involved in the early stages of RA, widespread dysregulation of non-antigen-specific cellular responses--including aggressive growth of fibroblast-like synoviocytes (FLSs), proinflammatory cytokine production by macrophages, and activation of osteoclasts--likely underlies the chronic inflammatory stage of RA. Elucidation of the cellular responses that are central to the pathogenesis of RA could lead to the development of novel targeted therapies.

Imatinib mesylate (imatinib) is a tyrosine kinase inhibitor approved for the treatment of Bcr-Abl-expressing chronic myelogenous leukemias and c-Kit-expressing gastrointestinal stromal tumors [5,6]. Recent case reports describe the alleviation of RA symptoms in RA patients receiving imatinib for the treatment of these cancers [7-9], suggesting that tyrosine kinases are important in the pathogenesis of RA. Indeed, we and others have shown that imatinib ameliorates autoimmune arthritis in animal models of RA [10-12]. At micromolar concentrations, imatinib inhibits a narrow spectrum of tyrosine kinases, including c-Kit, platelet-derived growth factor receptor (PDGFR) α/β, Abl, Abl-related kinases, and c-Fms (also known as colony-stimulating factor receptor 1) [13-15]. We previously demonstrated that micromolar concentrations of imatinib abrogated multiple pathways implicated in RA pathogenesis, including production of proinflammatory cytokines by synovial macrophages, proliferation of FLSs, production of TNF by mast cells, and proliferation of, and antibody production by, B cells [12]. These effects were associated with inhibition of c-Fms activation in synovial macrophages, of PDGFR activation in FLSs, and of c-Kit activation in mast cells. Still unknown are the relative contribution of these kinases and their associated cellular responses to the pathogenesis of RA. Elucidation of the kinases central to pathogenesis would enable the development of highly specific inhibitors with an improved therapeutic index for the treatment of RA.

Accumulating evidence underscores the importance of monocyte lineage cells in the chronic inflammatory stage of RA. Upon migration to tissues, monocytes differentiate into macrophages and osteoclasts, which perform several homeostatic functions [16,17]. In addition to their role in immune defense, macrophages clear cell debris and participate in tissue remodeling following an inflammatory response. Osteoclasts play a key role in bone remodeling by resorbing bone, and under physiological conditions, their activity is tightly coordinated with that of osteoblasts, which are responsible for forming bone [18]. In RA, monocyte lineage cells are aberrantly activated: an increase in macrophage infiltration of the synovium promotes inflammation via the production of TNF and other proinflammatory cytokines, and an increase in osteoclast activity promotes erosion of bone [19].

Development and proliferation of monocyte lineage cells are mediated by c-Fms [17], a member of the PDGFR family of tyrosine kinases. The c-Fms ligand macrophage colony-stimulating factor (M-CSF) is produced predominantly by FLSs, T cells, and endothelial cells, and its expression is upregulated in these cells in RA [20,21]. Recently, interleukin-34 (IL-34) was identified as a second ligand for c-Fms [22]. Although c-Fms has been implicated in RA, prior studies have not fully defined the cellular mechanisms by which c-Fms modulates autoimmune arthritis. Here, we dissect the role of c-Fms, demonstrating that c-Fms signaling promotes the formation and activation of macrophages and osteoclasts. These findings reveal the relevance of c-Fms to specific cellular processes important in the pathogenesis of RA. Furthermore, we demonstrate that a specific small-molecule inhibitor of c-Fms is effective in treating arthritis in multiple mouse models of RA.

## Materials and methods

### Small-molecule inhibitors and antibodies

**Figure 1:**
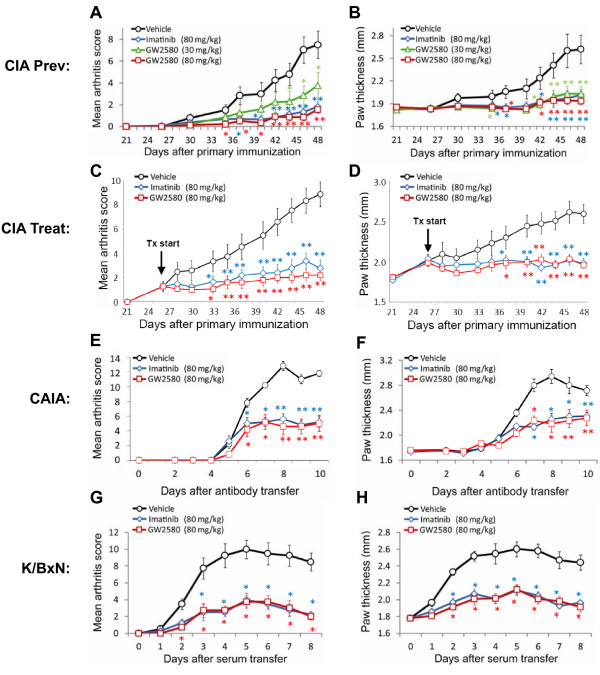
**c-Fms inhibition prevents and treats autoimmune arthritis**. Clinical arthritis scores (left panels) and paw thickness measurements (right panels) of arthritic mice treated with imatinib or GW2580. **(a, b) **Collagen-induced arthritis (CIA) prevention studies in DBA/1 mice; administration of vehicle (n = 12), 30 mg/kg GW2580 (n = 12), 80 mg/kg GW2580 (n = 12), or 80 mg/kg imatinib (n = 12) started 1 day before induction of CIA. **(c, d) **CIA treatment studies in DBA/1 mice; administration of vehicle (n = 15), 80 mg/kg GW2580 (n = 15), or 80 mg/kg imatinib (n = 15) started once CIA is established as indicated. **(e, f) **Anti-collagen antibody-induced arthritis (CAIA) prevention studies in BALB/c mice; administration of vehicle (n = 5), 80 mg/kg GW2580 (n = 5), or 80 mg/kg imatinib (n = 5) started 1 day before transfer of anti-collagen type II antibodies. **(g, h) **K/BxN prevention studies in BALB/c mice; administration of vehicle (n = 5), 80 mg/kg GW2580 (n = 5), or 80 mg/kg imatinib (n = 5) started 1 day before transfer of K/BxN serum. The data shown in (a-h) are representative of three independent experiments. Values are the mean ± standard error of the mean for the representative experiment shown. *P < 0.05, **P < 0.01 compared with vehicle-treated mice.

**Figure 2:**
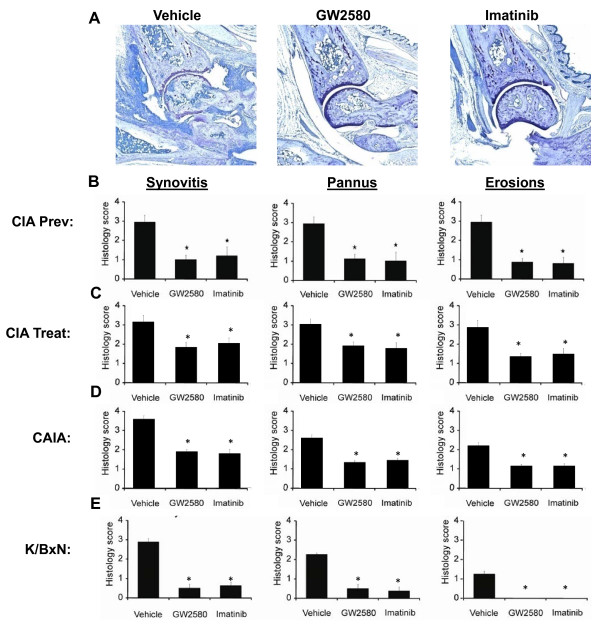
**c-Fms inhibition reduces synovitis, pannus formation, and joint erosion in autoimmune arthritis**. **(a) **Representative Toluidine blue-stained joint sections from DBA/1 mice in the collagen-induced arthritis (CIA) prevention study. Images are shown at × 100 magnification and are representative of at least two independent experiments. Histopathologic scores for synovitis, pannus formation, and joint erosion in **(b) **DBA/1 mice with CIA in the prevention study (vehicle, n = 10; 80 mg/kg GW2580, n = 10; 80 mg/kg imatinib, n = 10), **(c) **DBA/1 mice with CIA in the treatment study (vehicle, n = 8; 80 mg/kg GW2580, n = 8; 80 mg/kg imatinib, n = 8), **(d) **BALB/c mice with anti-collagen antibody-induced arthritis (CAIA) (vehicle, n = 5; 80 mg/kg GW2580, n = 5; 80 mg/kg imatinib, n = 5), and **(e) **BALB/c mice with K/BxN serum transfer arthritis (vehicle, n = 5; 80 mg/kg GW2580, n = 5; 80 mg/kg imatinib, n = 5). The data shown are representative of at least two independent experiments. Values are the mean ± standard error of the mean. *P < 0.05 compared with vehicle-treated mice.

**Figure 3:**
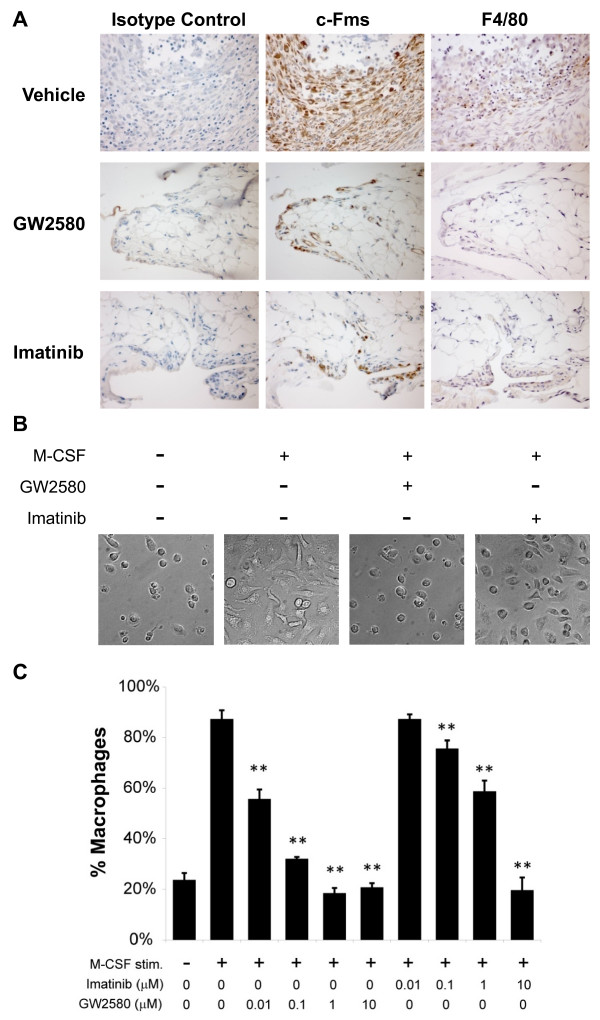
**c-Fms inhibition blocks macrophage differentiation and joint infiltration**. **(a) **Representative immunohistochemistry images of sections of ankle joint tissue from DBA/1 mice treated with vehicle, GW2580, or imatinib in a collagen-induced arthritis prevention study. Joint sections were stained with antibodies against total c-Fms, the macrophage marker F4/80, or antibody isotype controls. Images are shown at × 400 magnification and are representative of at least three independent experiments. **(b, c) **Differentiation to macrophages. Bone marrow cells from naïve BALB/c mice were treated with macrophage colony-stimulating factor (M-CSF) alone for 5 days to promote monocyte maturation and then incubated with (+) or without (-) M-CSF for an additional 48 hours in the presence of GW2580 or imatinib, as indicated. (b) Representative inverted microscopic images of untreated monocytes (left panel) and M-CSF-treated monocytes ± GW2580 or imatinib. (c) The percentage of macrophages in untreated or M-CSF-treated cultures in the presence of 0 to 10 μM GW2580 or imatinib was determined with an assay that detects α-naphtyl acetate esterase activity, coupled with fluoride inhibition. **P < 0.01.

**Figure 4:**
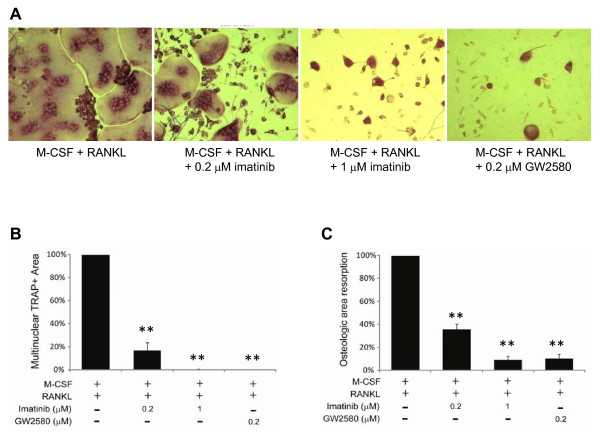
**c-Fms inhibition blocks osteoclast differentiation**. Bone marrow cells from naïve BALB/c mice were treated with macrophage colony-stimulating factor (M-CSF) alone for 24 hours and then transferred to plates with either dentine disks **(a, b) **or osteologic disks **(c) **and treated with M-CSF and receptor activator of nuclear factor-kappa B ligand (RANKL) ± GW2580 or imatinib. (a) Representative images showing reduction in tartrate-resistant acid phosphatase-positive (TRAP^+^) cell numbers following treatment with imatinib or GW2580. For quantification, the dentine disk area that stained positive for TRAP^+ ^multinucleated cells (b) and the degree of pit formation in osteologic disks (c) are expressed as a percentage of the area stained or of the pit formation detected following treatment with M-CSF and RANKL. The data shown are representative of at least two independent experiments. Values are the mean ± standard error of the mean. **P < 0.01 compared with cells treated with M-CSF and RANKL alone (b, c).

**Figure 5:**
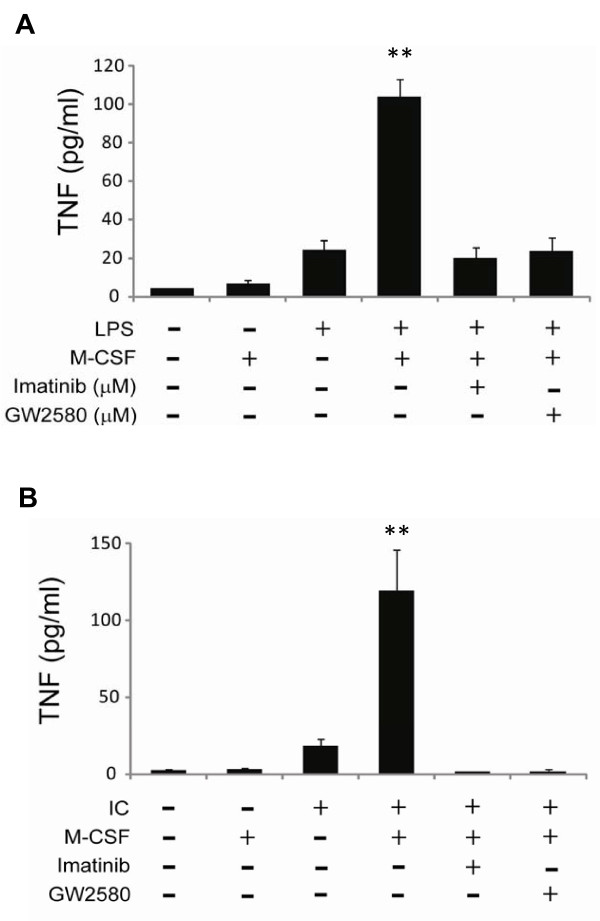
**c-Fms signaling primes macrophage response to lipopolysaccharide (LPS) or immune complex stimulation**. Fully differentiated, bone marrow-derived macrophages pretreated with macrophage colony-stimulating factor (M-CSF) for 3 hours in the absence or presence of 5 μM GW2580 or imatinib followed by stimulation with **(a) **low-dose LPS (1 ng/mL) or **(b) **FcRgII/III cross-linking (20 μg/mL plate-bound 2.4G2 antibody). After 24 hours of culture, tumor necrosis factor (TNF) in the supernatants was measured by enzyme-linked immunosorbent assay. Values are the mean ± standard error of the mean. **P < 0.01 compared with unprimed stimulated cells without inhibitor. Results are representative of at least three independent experiments. IC, immune complex.

**Figure 6:**
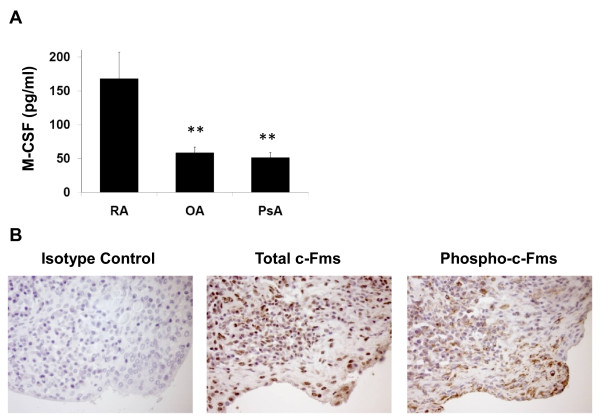
**Macrophage colony-stimulating factor (M-CSF), total c-Fms, and phospho-c-Fms are upregulated in human rheumatoid arthritis (RA) synovium**. **(a) **Levels of M-CSF in synovial fluid from patients with RA (n = 14), osteoarthritis (OA) (n = 15), or psoriatic arthritis (PsA) (n = 12) were measured by Luminex bead-based arrays. Values are the mean ± standard error of the mean. **P < 0.01 compared with RA samples. **(b) **Representative immunohistochemical images of RA synovium stained with antibodies against c-Fms, phospho-c-Fms, or isotype controls.

In the *in vitro *studies, we used imatinib mesylate that was chemically synthesized and confirmed to be more than 98% pure by the Organic Synthesis Core Facility at Memorial Sloan-Kettering Cancer Center (New York, NY, USA). In the *in vivo *studies, we used imatinib mesylate tablets (Stanford Inpatient Pharmacy Services, Palo Alto, CA, USA), which were ground and resuspended in the vehicle. GW2580 provided by GlaxoSmithKline (Uxbridge, Middlesex, UK) was used in the studies on prevention of arthritis (Figures 1 and 2). GW2580 purchased from Calbiochem (San Diego, CA, USA) and GW2580 chemically synthesized and confirmed to be more than 99% pure by SRI International (Menlo Park, CA, USA) were used in the studies on the treatment of arthritis (Figures 1 and 2), the experiments shown in Figures 3, 4, 5 and 6, and the experiments shown in Additional file 1. Anti-c-Fms, anti-phospho-c-Fms, and isotype control antibodies were from Santa Cruz Biotechnology, Inc. (Santa Cruz, CA, USA).

### IC_50 _determination

c-Kit and Abl kinase activity in the presence or absence of small-molecule inhibitors was determined by using HTScan kinase assay kits (Cell Signaling Technology, Inc., Danvers, MA, USA) coupled with europium-labeled DELFIA assays (PerkinElmer, Waltham, MA, USA), and counts were measured by time-resolved fluorescence (PerkinElmer) in accordance with the protocols of the manufacturer. To assess c-Fms activity, we incubated human peripheral blood mononuclear cells with 20 ng/mL M-CSF in the presence or absence of small-molecule inhibitors and determined the percentage of macrophages, as described below. To assess PDGFR activity, we isolated human FLSs as previously described [12], stimulated them for 72 hours with 20 ng/mL PDGF-bb in the presence of small-molecule inhibitors, pulsed them with 1 μCi [^3^H] thymidine (ICN Pharmaceuticals, Costa Mesa, CA, USA) for the final 18 hours of the stimulation, and used a Betaplate scintillation counter (PerkinElmer) to quantify the radioactivity incorporated. Scintillation counts were used to generate nonlinear regression dose-response curves for each small-molecule inhibitor, and IC_50_s (half inhibitory concentrations) were determined by using Prism software (GraphPad Software, Inc., San Diego, CA, USA).

### Synovial fluid and tissue samples from patients with arthritis

Human synovial fluid and synovial tissue samples were collected from RA, osteoarthritis (OA), and psoriatic arthritis (PsA) patients who met the American College of Rheumatology criteria. Samples were collected in accordance with protocols approved by the Stanford University Institutional Review Board after procurement of informed consent.

### Models of autoimmune arthritis

Six- to eight-week-old male DBA/1 mice and female BALB/c mice were purchased from The Jackson Laboratory (Bar Harbor, ME, USA) and housed at Stanford University under protocols approved by the Stanford University Committee of Animal Research and in accordance with National Institutes of Health guidelines. Collagen-induced arthritis (CIA) in DBA/1 mice was induced and scored as previously described [23]. Briefly, DBA/1 mice were immunized by intradermal injection of 100 μg/mouse bovine collagen type II (CII) (Chondrex, Inc., Redmond, WA, USA) emulsified in complete Freund's adjuvant (CFA) containing 250 μg/mouse heat-killed *Mycobacterium tuberculosis *H37Ra (Becton, Dickinson and Company, Franklin Lakes, NJ, USA). Twenty-one days after immunization, mice were given a subcutaneous boost injection (at the base of the tail) of 100 μg/mouse bovine CII emulsified in incomplete Freund's adjuvant (IFA). In BALB/c mice, anti-collagen antibody-induced arthritis (CAIA) was induced by intravenous injection of 1 mg of Arthrogen monoclonal antibody blend (Chondrex, Inc.) followed by 25 μg of lipopolysaccharide (LPS) (Chondrex, Inc.) 3 days later. K/BxN arthritis was induced in BALB/c mice by intraperitoneal (i.p.) injection of 1 μL of K/BxN serum per 1 g of mouse weight, followed 48 hours later by i.p. injection of 0.5 μL of K/BxN serum per 1 g of mouse weight. Arthritis severity was evaluated according to the following visual scoring system: 0 = no swelling or erythema; 1 = mild swelling and erythema of digits or paw; 2 = moderate swelling and erythema confined to the area distal to the mid-paw; 3 = more-pronounced swelling and erythema extending to the ankle; 4 = severe swelling, erythema, and joint rigidity of the ankle, foot, and digits. Each limb was assigned a score of 0 to 4, with a maximum possible score of 16 for each mouse. Paw thickness was determined by measuring the thickness of both hind paws with 0- to 10-mm calipers and calculating the mean of the two measurements.

### *In vivo *dosing with small-molecule inhibitors

For administration *in vivo*, GW2580 and imatinib were diluted in 0.5% hydroxypropylmethylcellulose and 0.05% Tween-80 solution. GW2580 and imatinib were delivered by oral gavage twice daily at the specified doses, starting 1 day before immunization in the CIA prevention studies, following arthritis development (average visual arthritis score of 2) in the CIA treatment studies, and 1 day before antibody transfer in the CAIA or K/BxN arthritis studies. Dosing was continued for the duration of the experiment. Administration of vehicle had no effect on the onset or severity of arthritis in mice.

### Histological evaluation

Hind limbs from mice with autoimmune arthritis were fixed and decalcified in CalEx II (Fischer Scientific, Pittsburgh, PA, USA) for 3 days before being paraffin-embedded. Histological assessment of arthritis severity was made by blinded evaluation of Toluidine blue-stained joint sections in accordance with a previously described scoring system: 0 = normal; 1 = mild inflammation, mild hyperplasia of the synovial lining layer, and mild cartilage destruction without bone erosion; 2 to 4 = increasing degrees of inflammatory cell infiltrates, synovial lining hyperplasia and pannus formation, and cartilage and bone destruction [24].

### Immunohistochemistry

Sections of paraffin-embedded synovium from RA patients and decalcified joint tissue from mice with autoimmune arthritis were deparaffinized, rehydrated, and subjected to antigen retrieval as described previously [25,26].

### Macrophage differentiation

Bone marrow cells were harvested from BALB/c mice and monocyte lineage cells were generated according to standard procedures [27]. After 4 to 5 days of culture, bone marrow-derived monocytes were incubated for 48 hours with 20 ng/mL M-CSF (PeproTech, Rocky Hill, NJ, USA) in the presence of 0 to 10 μM GW2580 or imatinib. To distinguish between monocytes and macrophages, we performed an α-napthyl acetate esterase assay, coupled with fluoride inhibition, in accordance with the protocol of the manufacturer (Sigma-Aldrich). At least 100 monocytes and macrophages were counted in triplicate for each experimental condition, and data are expressed as a percentage of macrophages in culture.

### Osteoclast differentiation

Twenty-four hours after their isolation from BALB/c mice, undifferentiated bone marrow cells were transferred to dentine disks (Immunodiagnostic Systems, Scottsdale, AZ, USA) or osteologic disks (BD Biosciences, San Jose, CA, USA) and cultured for 6 days in the presence of 50 ng/mL M-CSF and 50 ng/mL receptor activator of nuclear factor-kappa-B ligand (RANKL) (PeproTech) together with 0 to 5 μM small-molecule inhibitor. To identify multinucleated, tartrate-resistant acid phosphatase-positive (TRAP^+^) osteoclasts, we stained cells cultured on dentine disks with the acid phosphatase leukocyte kit (Sigma-Aldrich). ImageJ software was used to determine the dentine disk area that stained positive for TRAP^+ ^multinucleated cells. Pit formation was assessed by measuring the removal of surface film on osteologic disks with the Bioquant Osteo II image quantification system (Bioquant Image Analysis Corporation, Nashville, TN, USA).

### Macrophage priming

Bone marrow cells were harvested from BALB/c mice and macrophages were generated as previously described [27]. Macrophages were cultured overnight in complete RPMI media in the absence of M-CSF and then incubated for 3 hours in the presence of 0 to 50 ng/mL M-CSF and 0 to 5 μM small-molecule inhibitor, as described above. After 3 hours, cells were stimulated with 1 ng/mL LPS (Sigma-Aldrich) or 20 μg/mL plate-bound rat anti-mouse 2.4G2 (BD Biosciences) for 24 hours, as previously described [28], and supernatants were harvested for cytokine analysis by enzyme-linked immunosorbent assay (ELISA).

### T-cell stimulation

Splenocytes from CIA mice treated chronically with 80 mg/mL GW2580, 80 mg/mL imatinib, or vehicle were stimulated for 72 hours with 20 μg/mL whole, denatured bovine CII (Chondrex, Inc.). One microcurie of [^3^H] thymidine (ICN Pharmaceuticals) was added for the final 18 hours of culture, and radioactivity incorporation was quantified by using a Betaplate scintillation counter. Supernatants after 72 hours were harvested for cytokine analysis by ELISA.

### Statistics

Visual arthritis scores, paw thicknesses, and histology scores were compared by the Mann-Whitney *U *test with GraphPad InStat Version 3.0 (GraphPad Software, Inc.). Differences in arthritis scores were determined by the Fisher test with Analyse-it plug-in software (Analyse-it Software, Ltd., Leeds, UK) for Excel (Microsoft Corporation, Redmond, WA, USA). Macrophage differentiation, osteoclast differentiation, macrophage priming, and cytokine level were compared by unpaired *t *tests with GraphPad InStat Version 3.0 (GraphPad Software, Inc.).

## Results

### c-Fms inhibition prevents and treats autoimmune arthritis

To determine whether specific inhibition of c-Fms provides benefit in autoimmune arthritis, we explored the effects of GW2580 in several distinct models of RA and compared them with the effects of imatinib. Imatinib inhibits c-Kit, Abl, PDGFR, and c-Fms with IC_50_s of 0.1, 0.25, 0.1, and 1.4 μM, respectively. On the basis of published pharmacokinetic profiles [12], imatinib was administered to mice orally, twice daily at a dose of 80 mg/kg. GW2580 was administered to mice orally, twice daily at doses of 30 or 80 mg/kg. Previous pharmacokinetic studies in mice have determined that oral administration of 80 mg/kg GW2580 yields a maximal plasma concentration of 5.6 μM [29]. To determine the IC_50 _of GW2580 for the kinases c-Kit and Abl, we used cell-free kinase assays with time-resolved fluorescence. The IC_50_s were 73.5 μM for Abl (Additional file 1a) and greater than 100 μM for c-Kit (Additional file 1b) and concentrations significantly above the maximal plasma concentrations of GW2580 achieved in mice receiving 80 mg/kg GW2580. Using cell-based assays, we showed that GW2580 potently inhibits c-Fms (IC_50 _= 0.01 μM; Additional file 1c) and can inhibit PDGFR only at supraphysiological concentrations (IC_50 _= 12.1 μM; Additional file 1d). Thus, dosing of mice with GW2580 at a concentration of 80 mg/kg or less should inhibit c-Fms but not Abl, c-Kit, or PDGFR. Indeed, in a cell-free assay that measures the specificity of small-molecule inhibitors, GW2580 at 10 μM abolished c-Fms activity and did not cross-react with nearly 200 other kinases [30].

CIA was induced by injection of DBA/1 mice with bovine CII emulsified in CFA, followed 21 days later by a boost injection of CII emulsified in IFA. When imatinib dosing was initiated 1 day before the induction of CIA, it significantly reduced the severity of arthritis (Figure 1a, b), in agreement with our previous findings [12]. Likewise, mean arthritis scores and paw thickness measurements were significantly lower in mice dosed prophylactically with 30 or 80 mg/kg GW2580 compared with mice dosed with vehicle. GW2580 was as efficacious as imatinib in preventing the development of arthritis. Furthermore, when the kinase inhibitors were administered after the induction of arthritis, both GW2580 and imatinib significantly inhibited the progression of arthritis (Figure 1c, d). Mice were sacrificed between days 48 and 50 as this represents the peak of synovitis and inflammation. In the CIA experiments presented, all mice developed arthritis by the time the experiment was terminated (100% incidence).

Imatinib has been shown to ameliorate CAIA [10]. We performed experiments to determine whether specific inhibition of c-Fms would yield a similar benefit in CAIA. We induced CAIA by injecting BALB/c mice with 1 mg of anti-collagen antibodies, followed by 25 μg of LPS 3 days later. Administration of GW2580 or imatinib was started 1 day before the transfer of antibodies. All CAIA mice developed arthritis by day 6 after antibody transfer (100% incidence). Arthritis was significantly less severe in CAIA mice treated with the c-Fms-specific inhibitor GW2580 compared with vehicle-treated CAIA mice (Figure 1e, f). The course of arthritis in GW2580-treated CAIA mice mirrored that in imatinib-treated CAIA mice.

We induced K/BxN arthritis in BALB/c mice by transferring 1 μL of serum/g of mouse weight, followed by 0.5 μL of serum/g of mouse weight 48 hours later. Administration of GW2580 or imatinib was initiated 1 day before the transfer of serum. All K/BxN mice developed arthritis by day 4 after serum transfer (100% incidence). Arthritis was significantly less severe in K/BxN mice treated with GW2580 or imatinib compared with vehicle-treated K/BxN mice (Figure 1g, h).

### c-Fms inhibition reduces histopathologic severity in autoimmune arthritis

Histological analysis was performed on hind paws harvested from mice treated with 80 mg/kg GW2580, 80 mg/kg imatinib, or vehicle in the studies described above. Representative images of Toluidine blue-stained joint tissue sections from GW2580-, imatinib-, and vehicle-treated mice in the CIA prevention studies are presented (Figure 2a). Histopathologic evaluation by an investigator blinded to treatment groups for synovitis, formation of pannus, and erosion of cartilage and bone in paws derived from mice in CIA prevention (Figure 2b, n = 10 mice per group, with both hind paws from each mouse scored), CIA treatment (Figure 2c, n = 8 mice per group, with both hind paws from each mouse scored), CAIA (Figure 2d, n = 5 mice per group, with both hind paws for each mouse scored), and K/BxN (Figure 2e, n = 5 mice per group, with both hind paws of each mouse scored) studies. In contrast, these histological indices of arthritis were significantly reduced in paws from GW2580- or imatinib-treated mice in all four models of autoimmune arthritis.

### c-Fms inhibition does not modulate T-cell function *in vivo*

Because imatinib has been shown to modulate T-cell function, we investigated whether specific inhibition of c-Fms with GW2580 affects T-cell function. Splenocytes harvested from CIA mice treated with 80 mg/kg GW2580, 80 mg/kg imatinib, or vehicle in the prevention studies were stimulated *ex vivo *with heat-denatured whole CII, and [^3^H] thymidine incorporation was used as a surrogate marker of T-cell proliferation. Cells harvested from vehicle- and GW2580-treated CIA mice proliferated extensively in response to CII, whereas cells harvested from imatinib-treated CIA mice exhibited a significantly reduced response (Additional file 2). In addition, splenocytes derived from imatinib-treated CIA mice stimulated with CII demonstrated significantly reduced production of the proinflammatory cytokines TNF and interferon-gamma compared with splenocytes derived from vehicle- or GW2580-treated CIA mice. There were no differences in IL-10 production in these same cell populations stimulated with CII. Thus, imatinib modulates T-cell function in vivo, whereas GW2580 does not.

### c-Fms inhibition blocks differentiation of monocyte cells into macrophages

To determine the effects of imatinib and GW2580 on macrophage infiltration of mouse joints, we assessed levels of total c-Fms and the macrophage-specific marker F4/80 in joint sections derived from CIA mice used in the prevention studies. We found that joints from CIA mice treated with vehicle exhibited marked expression of c-Fms protein, which colocalized with macrophages (Figure 3a). In contrast, in joints from CIA mice treated with 80 mg/kg GW2580 or 80 mg/kg imatinib, both c-Fms protein expression and macrophage infiltration were reduced.

To determine whether c-Fms inhibition affects the formation of macrophages, we isolated bone marrow cells from naïve BALB/c mice, treated them with M-CSF for 5 days to promote the maturation of monocytes, and cultured them for an additional 48 hours with M-CSF to promote their differentiation into macrophages, in the presence or absence of small-molecule inhibitors. Monocytes treated with M-CSF alone displayed a characteristic macrophage phenotype, including extension of multipolar processes and presence of heterogeneous cytoplasmic vacuoles and inclusion bodies (Figure 3b). In contrast, monocytes incubated with media alone and M-CSF-stimulated monocytes treated with 1 μM GW2580 morphologically resembled undifferentiated monocytes. Treatment of monocytes with M-CSF and 1 μM imatinib resulted in a heterogeneous mix of cells, of which some morphologically resembled monocytes and others resembled macrophages.

To confirm the effects of the c-Fms inhibitors on macrophage formation, we applied an assay for the detection of α-naphthyl acetate esterase activity, coupled with fluoride inhibition, to the cell culture system described above. In this assay, the granules of differentiated macrophages stain, whereas monocytes remain unstained [31]. Monocytes were generated as described above, cultured for 48 hours with M-CSF in the presence of 0 to 10 μM GW2580 or imatinib, and then fixed and stained for α-naphthyl acetate esterase activity. Approximately 20% of monocytes cultured in media alone formed macrophages; the addition of M-CSF increased the percentage of macrophages to nearly 90% of the total cell population (Figure 3c). Imatinib reduced macrophage formation in a dose-dependent manner. GW2580 suppressed macrophage formation at lower concentrations than imatinib, in keeping with GW2580 being the more potent c-Fms inhibitor. There were no differences in apoptosis between untreated cells and cells treated with imatinib or GW2580 (data not shown).

### c-Fms inhibition blocks the differentiation of monocytes into osteoclasts

We performed experiments to determine whether GW2580 could suppress the formation of osteoclasts. Bone marrow cells were isolated from naïve BALB/c mice, and after 24 hours in culture with M-CSF, the suspension cells were transferred to plates containing dentine or osteologic discs and were co-cultured with M-CSF and RANKL in the presence or absence of GW2580 or imatinib. Treatment with M-CSF and RANKL led to marked formation of large TRAP^+ ^multinucleated cells, which are indicative of osteoclasts. For each treatment condition, the dentine disk area that stained positive for TRAP^+ ^multinucleated cells was calculated by ImageJ software and expressed as a percentage of the area stained positive for TRAP^+ ^following treatment with M-CSF and RANKL alone. Both GW2580 and imatinib significantly reduced the formation of multinucleated TRAP^+ ^osteoclasts, albeit with different potencies (inhibition achieved with 0.2 μM GW2580 was comparable to that achieved with 1 μM imatinib) (Figure 4a, b). Because osteoclasts erode bone in RA joints, we next examined the formation of pits in osteologic disks, an indication of the ability of osteoclasts to resorb bone. We found that treatment of cells with imatinib (0.2 or 1 μM) resulted in a dose-dependent reduction in pit formation and that treatment with 0.2 μM GW2580 blocked pit formation (Figure 4c).

### c-Fms activation primes macrophage tumor necrosis factor production in response to lipopolysaccharide or immune complex stimulation

Whether c-Fms activation promotes anti-inflammatory or proinflammatory activity in fully differentiated macrophages is controversial [32,33]. To explore the role of c-Fms in macrophage production of the proinflammatory cytokine TNF, we performed priming studies with fully differentiated macrophages derived from bone marrow cells of BALB/c mice. Macrophages primed with M-CSF for 3 hours in the presence or absence of 5 μM GW2580 or imatinib were stimulated with 1 ng/mL LPS for 24 hours, and TNF protein in the cell culture supernatants was then measured by ELISA. Cells cultured in media alone or M-CSF alone produced very little TNF (Figure 5a). Low-dose LPS stimulated TNF production, which was significantly increased in macrophages primed with M-CSF for 3 hours before the addition of LPS. GW2580 or imatinib at 5 μM (a clinically relevant concentration) reduced TNF production to levels detected in cells treated with LPS alone.

Although LPS is routinely used in experimental settings for the stimulation of proinflammatory cytokine production by macrophages, it is not a true trigger for the production of TNF in RA. Important physiological triggers of inflammation in RA are immune complexes that bind and activate Fc receptors [34]. To examine the role of c-Fms in TNF production elicited by immune complexes, we used the FcR-specific antibody 2.4G2 as a surrogate for immune complex-mediated Fc activation. Stimulation of macrophages with 2.4G2 alone for 24 hours elicited a small increase in TNF production (Figure 5b). Macrophages that were first primed with M-CSF for 3 hours and then stimulated with 2.4G2 produced substantial amounts of TNF. Co-culturing of cells with 5 μM GW2580 or imatinib blocked the M-CSF- and 2.4G2-induced increase in TNF production.

### Levels of total c-Fms, phospho-Fms, and M-CSF in rheumatoid arthritis patients

To demonstrate involvement of the M-CSF/c-Fms axis in human RA, we used Luminex bead arrays to determine M-CSF protein levels in synovial fluid derived from patients with RA, OA, or PsA. M-CSF levels were significantly higher in human RA synovial fluid than in OA or PsA synovial fluid (Figure 6a). We also assessed levels of total c-Fms and phosphorylated c-Fms in human RA synovium and found that both expression and activation of c-Fms are high (Figure 6b).

## Discussion

Imatinib studies to date have suggested that c-Fms, PDGFR, c-Kit, and/or Abl tyrosine kinase pathways may contribute to the pathogenesis of RA, but the dominant pathways and cellular mechanisms involved remain unclear [10-12]. Prior studies have not addressed the specific cellular mechanisms by which c-Fms mediates autoimmune arthritis. The studies provided herein suggest that c-Fms-mediated differentiation and activation of macrophages and osteoclasts play a central role in the pathogenesis of autoimmune arthritis. Furthermore, the c-Fms-specific inhibitor GW2580 was as efficacious as imatinib in the treatment of autoimmune arthritis.

In an adjuvant arthritis model in rats, GW2580 suppressed bone destruction but did not affect joint inflammation [30]. Here, we show that GW2580 inhibits both bone erosion and joint inflammation in multiple mouse models of RA, indicating that c-Fms plays a critical role in regulating inflammatory arthritis. Reported disparities in the effects of candidate therapeutic compounds on inflammatory arthritis may be due to differences in the RA models used, none of which encompasses all of the features of RA; therefore, candidate therapeutics should ideally be tested in multiple models of RA [35]. We investigated the effects of GW2580 in three distinct models of RA. Induction of arthritis in the CIA model is dependent on adaptive immune responses, whereas induction in the CAIA and K/BxN models--antibody transfer models that bypass the requirement for T and B cells--is dependent solely on innate immune responses. Inhibition of c-Fms reduced arthritis severity in both types of RA models, indicating that c-Fms is integral to the pathogenesis of autoimmune arthritis. Furthermore, GW2580 reduced arthritis severity when administered either before the onset of arthritis or following the establishment of arthritis, suggesting that c-Fms plays a role in both the early and the chronic stages of autoimmune arthritis. Our data are consistent with previous findings demonstrating the importance of the c-Fms ligand M-CSF in CIA: exogenous M-CSF was shown to exacerbate CIA, whereas a neutralizing antibody against M-CSF reduced arthritis severity and M-CSF deficiency conferred resistance to CIA [36]. Recently, IL-34 was identified as a second ligand for c-Fms [22]; the role of IL-34-mediated stimulation of c-Fms in RA remains to be investigated.

M-CSF/c-Fms signaling drives the differentiation of monocytes into macrophages or osteoclasts, both of which contribute to synovial inflammation and joint destruction in RA. Using mouse models of autoimmune arthritis, we demonstrate that GW2580 blocks the formation of macrophages and osteoclasts *in vitro *and reduces macrophage infiltration of joints *in vivo*. Furthermore, we demonstrate that M-CSF and both total and activated c-Fms are highly expressed in the synovium of RA patients. Together, our data underscore the importance of the M-CSF/c-Fms signaling pathway in RA pathogenesis and suggest that inhibition of c-Fms ameliorates autoimmune arthritis by abrogating the differentiation of monocyte lineage cells.

An increase in osteoclast numbers in RA leads to pathogenic degradation of bone [37]. Both the M-CSF/c-Fms and the RANKL/RANK signaling pathways are required for formation of osteoclasts. Results from a recent phase II trial demonstrated that blockade of RANKL with denosumab decreased structural damage, including bone erosions, in patients with RA; however, it had no effect on the American College of Rheumatology response criteria, DAS28 (disease activity score using 28 joint counts) criteria, or RA flares [38]. Similarly, preclinical studies demonstrated that RANKL inhibition mitigated bone erosions without improving clinical parameters of disease in autoimmune arthritis [39]. Thus, in the treatment of autoimmune arthritis, inhibiting RANKL is not as efficacious as inhibiting c-Fms. We propose that inhibition of c-Fms is more efficacious because c-Fms is crucial to the formation of macrophages in addition to osteoclasts.

The clinical improvement following c-Fms inhibition in our autoimmune arthritis models was rapid. Such rapidity of the response cannot be attributed to effects on differentiation of monocyte lineage cells, a process that occurs over several days, nor can it be attributed to effects on T cells as splenocytes from GW2580-treated CIA mice exhibited activity similar to splenocytes from vehicle-treated CIA mice. This is in contrast to results from studies using the c-Fms inhibitor Ki20227, in which Ki20227-mediated suppression of CIA was associated with a reduction in splenocyte activity [40]. However, Ki20227 inhibits vascular endothelial growth factor receptor-II (VEGFR-II) in addition to c-Fms, and its selectivity has not been extensively evaluated [41]; it is possible that Ki20227 inhibits additional kinases that are important in T-cell signaling. What then is the basis for such a rapid response to c-Fms inhibition? A crucial role for macrophages in the development of RA is the production of TNF and other cytokines that promote inflammation [1,42]. We demonstrate that c-Fms activation primes macrophage TNF production such that macrophages produce much greater amounts of TNF upon Fc receptor stimulation, an important trigger of synovitis in RA [34]. Thus, we propose that blockade of a c-Fms-mediated priming effect on macrophage TNF production underlies the rapidity of the clinical response to c-Fms inhibition.

The CAIA and K/BxN models result in the formation of immune complexes that activate complement, resulting in the recruitment and activation of neutrophils and macrophages to produce TNF and other inflammatory mediators. As shown in Figure 5b, we demonstrate that specific Fms inhibition potently blocks TNF release in response to immune complexes. Thus, inhibition of TNF production from immune complex-stimulated macrophages by GW2580 likely represents a primary mechanism by which Fms inhibition provides benefit in CAIA and K/BxN arthritis.

Although our results indicate that c-Fms plays a key role in the pathogenesis of RA, they do not preclude contributions by other receptor tyrosine kinases. Mice in which c-Kit signaling is impaired--owing to a loss-of-function mutation in either the gene encoding c-Kit or the gene encoding the c-Kit ligand--are resistant to antibody-mediated arthritis [43,44]. Indeed, masitinib, a tyrosine kinase inhibitor that is more selective than imatinib for c-Kit, recently demonstrated favorable trends in an uncontrolled phase II trial [45]. However, masitinib potently inhibits the PDGFRα/β and Lyn kinases in addition to c-Kit, and thus the feasibility of treating RA by selectively inhibiting c-Kit remains to be explored. Furthermore, PDGFR signaling in FLSs and other cell types may also contribute to the development of autoimmune arthritis. Hyperproliferation of FLSs, which contribute to the formation of tumor-like pannus in RA joints, likely results in part from an increase in PDGFR expression and activity [46]. In addition, PDGFR signaling may promote synovitis in RA by inducing the production of proinflammatory cytokines by FLSs [47]. To date, however, assessment of the importance of PDGFR in autoimmune arthritis and RA has been hampered by the lethality of PDGFR_ or PDGFRβ deficiency in mice and the lack of small-molecule inhibitors that selectively target PDGFRα/β. Thus, the role of PDGFR in autoimmune arthritis awaits further clarification.

## Conclusions

We show that c-Fms plays a pivotal role in autoimmune arthritis by promoting the differentiation and priming of monocyte lineage cells. Selective c-Fms inhibition affords targeting of multiple pathogenic cell types and responses in autoimmune arthritis and represents a promising approach to the treatment of RA. Clinical trials are warranted to evaluate the efficacy and therapeutic index of c-Fms inhibitors in RA.

## Abbreviations

CAIA: anti-collagen antibody-induced arthritis; CFA: complete Freund's adjuvant; CIA: collagen-induced arthritis; CII: collagen type II; ELISA: enzyme-linked immunosorbent assay; FLS: fibroblast-like synoviocyte; IC_50_: half inhibitory concentration; IFA: incomplete Freund's adjuvant; IL: interleukin; i.p.: intraperitoneal; LPS: lipopolysaccharide; M-CSF: macrophage colony-stimulating factor; OA: osteoarthritis; PDGFR: platelet-derived growth factor receptor; PsA: psoriatic arthritis; RA: rheumatoid arthritis; RANKL: receptor activator of nuclear factor-kappa-B ligand; TNF: tumor necrosis factor; TRAP^+^: tartrate-resistant acid phosphatase-positive.

## Competing interests

The authors declare that they have no competing interests.

## Authors' contributions

RTP helped to design the experiments and interpret the data, contributed to the writing of the manuscript, and helped to perform the experiments and generate the datasets. WHR helped to design the experiments and interpret the data and contributed to the writing of the manuscript. AC, MM, EAS, QW, OS, CR, and PPH helped to perform the experiments and generate the datasets. TML contributed to the interpretation of the datasets and the writing of the manuscript. DML oversaw the immunohistochemistry studies along with presentation and interpretation of the immunohistochemistry data. All authors read and approved the final manuscript.

## Supplementary Material

Additional file 1 GW2580 potently inhibits c-Fms kinase and does not cross-react with other imatinib-targeted kinases at clinically relevant concentrations. **(a, b) **Cell-free kinase activity assay with time-resolved fluorescent readout for determination of the IC_50 _of imatinib and GW2580 for the kinases **(a) **Abl and **(b) **c-Kit. **(c) **Cell-based assay for determination of the IC_50 _of imatinib and GW2580 for c-Fms. Human peripheral blood mononuclear cells were treated with M-CSF in the presence of 0-10 μM GW2580 or imatinib for 48 hours. Macrophages were counted and values expressed relative to M-CSF treatment alone. **(d) **Cell-based assay for determination of the IC_50 _of imatinib and GW2580 for PDGFR. Fibroblast-like synoviocytes from a human RA patient were incubated with PDGF-bb in the presence of 0-30 μM GW2580 or imatinib. After 48 hours, FLS cultures were pulsed with [^3^H] thymidine for 18 hours. Values are expressed relative to PDGF-bb treatment. Data shown in **a-d **are representative of at least 2 independent experiments. Click here for file

Additional file 2 GW2580 does not modulate T-cell function *in vivo*. Splenocytes were harvested from DBA/1 mice with CIA and treated with GW2580, imatinib, or vehicle, and stimulated with 20 μg/ml heat-denatured, whole CII. [^3^H]thymidine incorporation was used to measure proliferation of CII-specific T cells. IFNγ, TNFα and IL-10 were measured in culture supernatants by ELISA. Values are the mean ± SEM. *P < 0.05 compared with stimulated cells from vehicle-treated CIA mice. Results are representative of 2 independent experiments. Click here for file
