# Supplementary figures and images for: c-Fms-mediated differentiation and priming of monocyte lineage cells play a central role in autoimmune arthritis
Source: Arthritis Res Ther. 2010 Feb 24;12(1):R32. doi: 10.1186/ar2940 (PMC2875666; doi:10.1186/ar2940)

**A**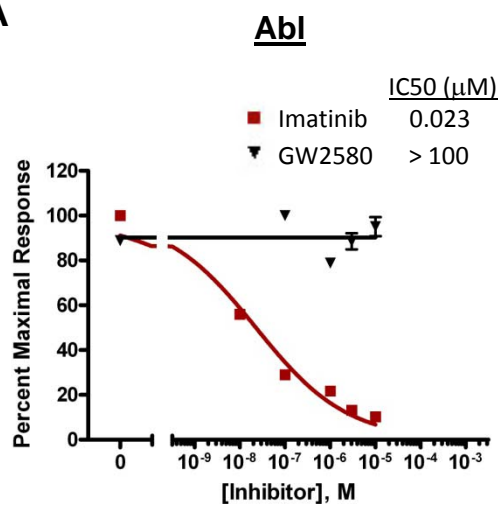**B**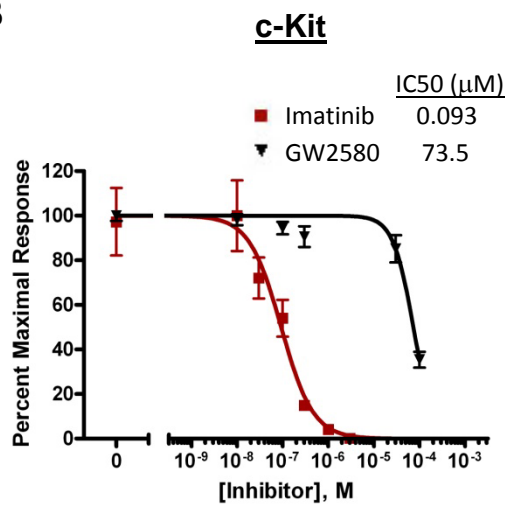**C**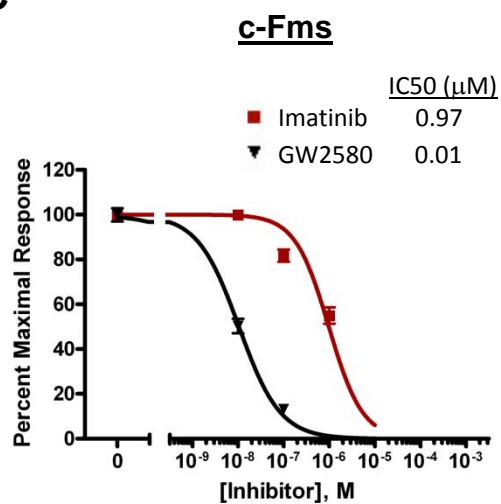**D**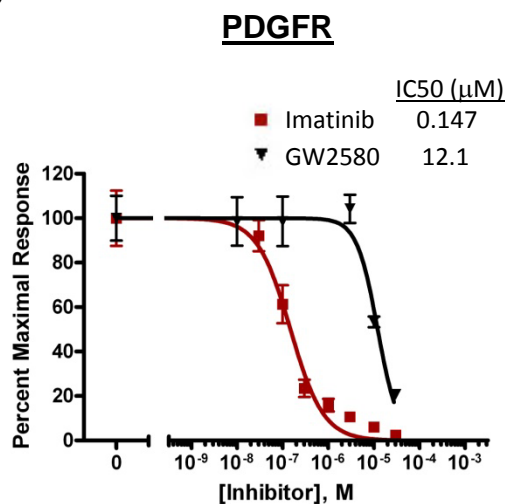

Additional File 1. *Paniagua et al*

Supplement: Additional file 1 — GW2580 potently inhibits c-Fms kinase and does not cross-react with other imatinib-targeted kinases at clinically relevant concentrations. (a, b) Cell-free kinase activity assay with time-resolved fluorescent readout for determination of the IC50 of imatinib and GW2580 for the kinases (a) Abl and (b) c-Kit. (c) Cell-based assay for determination of the IC50 of imatinib and GW2580 for c-Fms. Human peripheral blood mononuclear cells were treated with M-CSF in the presence of 0-10 μM GW2580 or imatinib for 48 hours. Macrophages were counted and values expressed relative to M-CSF treatment alone. (d) Cell-based assay for determination of the IC50 of imatinib and GW2580 for PDGFR. Fibroblast-like synoviocytes from a human RA patient were incubated with PDGF-bb in the presence of 0-30 μM GW2580 or imatinib. After 48 hours, FLS cultures were pulsed with [3H] thymidine for 18 hours. Values are expressed relative to PDGF-bb treatment. Data shown in a-d are representative of at least 2 independent experiments. [file ar2940-S1.PDF]

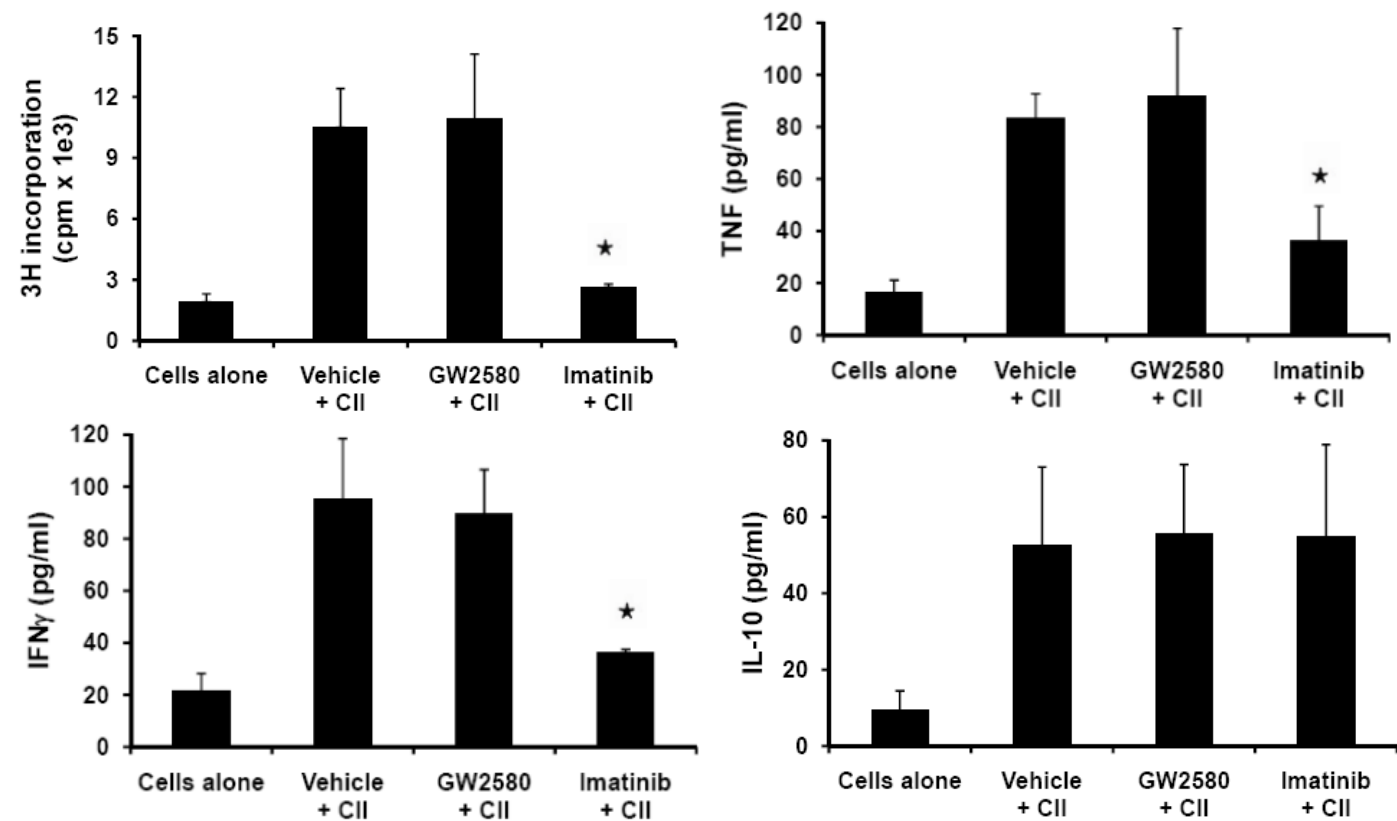

Additional File 2. Paniagua *et al*

Supplement: Additional file 2 — GW2580 does not modulate T-cell function in vivo. Splenocytes were harvested from DBA/1 mice with CIA and treated with GW2580, imatinib, or vehicle, and stimulated with 20 μg/ml heat-denatured, whole CII. [3H]thymidine incorporation was used to measure proliferation of CII-specific T cells. IFNγ, TNFα and IL-10 were measured in culture supernatants by ELISA. Values are the mean ± SEM. *P < 0.05 compared with stimulated cells from vehicle-treated CIA mice. Results are representative of 2 independent experiments. [file ar2940-S2.PDF]
